# Next Generation Influenza Vaccines: Looking into the Crystal Ball

**DOI:** 10.3390/vaccines8030464

**Published:** 2020-08-21

**Authors:** Carlos Alberto Guzmán

**Affiliations:** Department of Vaccinology and Applied Microbiology, Helmholtz Centre for Infection Research, Inhoffenstrasse 7, 38124 Braunschweig, Germany; CarlosAlberto.Guzman@helmholtz-hzi.de

**Keywords:** influenza, influenza vaccine, universal influenza vaccine, next generation influenza vaccine, egg-adaptation, T cell influenza vaccine, RNA influenza vaccine, original antigenic sin

## Abstract

Influenza infections are responsible for significant number of deaths and overwhelming costs worldwide every year. Vaccination represents the only cost-efficient alternative to address this major problem in human health. However, current vaccines are fraught by many limitations, being far from optimal. Among them, the need to upgrade vaccines every year through a time-consuming process open to different caveats, and the critical fact that they exhibit poorer efficacy in individuals who are at high risk for severe infections. Where are we? How can knowledge and technologies contribute towards removing current roadblocks? What does the future offer in terms of next generation vaccines?

Influenza viruses periodically cause large epidemics, which are associated with significant health care costs. Depending on the influenza season, between 290,000 and 650,000 people die yearly worldwide from influenza virus infection [1]. However, there are seasons in which the number of deaths is considerably higher, like in the 2016/2017 season. Despite the availability of antiviral drugs, the emergence of resistant strains, modest efficacy in severe influenza cases and/or a narrow therapeutic window [2] make vaccines still the most cost-efficient tool to fight seasonal and pandemic outbreaks. Accordingly, seasonal influenza vaccination is particularly recommended for those who are ≥60 years of age, as well as for persons with increased risks for severe influenza, such as pregnant women and individuals with comorbidities (e.g., chronic lung or heart diseases, diabetes). However, responsiveness to vaccination is often suboptimal in these groups at high-risk for severe infections [3,4,5].

The presence of a segmented viral genome and a low-fidelity RNA polymerase, together with the existence of viral zoonotic reservoirs, results in the accumulation of mutations over time (i.e., antigenic drift), as well as the sporadic emergence of viruses exhibiting major antigenic changes due to re-assorted genomes (i.e., antigenic shift). This rapid viral evolution promotes viral escape and renders necessary the revision of the influenza vaccine formulation every flu season. This represents a major manufacturing challenge for the vaccine industry, considering the currently existing technologies. The review by Harding and Heaton describe in detail the major current vaccine manufacturing approach based on virus replication in embryonated eggs [6]. The authors further analyzed the advantages and drawbacks of this approach, which allows producing over 1.5 billion doses every year [7]. They also benchmarked the two major alternatives exploited in approved vaccines, aimed at increasing the consistency of vaccine efficacy across seasons. On the one hand, the cell-based approaches, which not only eliminate the adverse events resulting from allergies to egg products and the constraints of egg-shortages, and reduce production times, but most importantly, reduce the risk of altered antigenicity as a result of the emergence of mutations during the adaptation to eggs [8]. It is important to highlight that mutations in the genes encoding the major vaccine antigens were also observed using this approach [9]. The authors also analyzed how the constraints of egg-produced vaccines affected their efficacy in recent seasons. On the other hand, they presented the pros and cons of protein-based vaccines produced using a Baculovirus-based approach, which eliminates the constraints resulting from relying on influenza virus replication. Nevertheless, practical considerations, like manufacturing capacity to serve the huge demand for influenza vaccines and considerably higher production costs still represent a bottleneck.

In their review article, the authors also presented a comprehensive summary of the current efforts to improve the influenza vaccines produced using standard existing technologies. In this regard, the use of adjuvants to improve the overall efficacy of vaccines for specific subpopulation groups is discussed, as well as the exploitation of nanotechnologies to increase the immunogenicity of protein-based vaccines. An elegant approach enables the engineering of recombinant viruses for vaccine production, which co-expresses the vaccine relevant hemagglutinin together with a helper hemagglutinin [10]. This strategy prevents the emergence of egg-adaptive mutations and reduces the overall production times. They also address the use of peptide-based vaccines as a potential alternative. Although promising, the presented drawbacks suggest that considerable time and effort will be required to make it a viable approach for implementation in the influenza vaccine field. The review by Clemens et al. further elaborates on the potential and limitations of harnessing T cells for the development of influenza vaccines [11]. In this particular context, animal studies support the potential of exploiting this strategy to generate universal or at least broader next generation vaccines providing heterosubtypic protection by stimulating long-lasting T cell resident memory cells. Nevertheless, this approach faces several major constraints. As described in the review, a universal vaccine based on peptides should mimic more closely the immune responses observed after natural infections by inducing both cross-reactive peripheral and tissue resident CD8 T cells in addition to cross-reactive antibodies and CD4 T follicular and helper cells. However, as described in the review, special vaccination strategies will be needed to promote the seeding of tissue resident memory T cells in the respiratory tract and prevent their attrition. Furthermore, universal protection will require addressing human leucocyte antigen (HLA)-restriction by the inclusion of appropriate peptides. Such vaccines should not only stimulate multiple cross-reactive epitope-specific T cells, but also encompass epitopes recognized by rare HLA types from minor ethnicities. Finally, current influenza vaccines are approved dependent on the accepted correlates for protection based on the stimulation of functional antibodies recognizing the hemagglutinin. Large-scale clinical studies will be needed to define similar robust correlates for a conceptually novel class of T cell influenza vaccines.

New disruptive technologies, such as the use of nucleic acid vaccines (e.g., DNA or RNA), which render unnecessary the production of proteins, can be also exploited to develop next generation influenza vaccines. In this regard, RNA vaccines represent a cutting-edge approach, which dramatically reduces production times, ensure the fidelity of the encoded sequences, and are insulated from shortages of eggs or the constraints attached to cell factories. This renders this technology particularly appealing for potential emerging influenza pandemics. Preclinical studies in different animal species and first-in-man studies also demonstrated the intrinsic value of this approach [12,13]. However, these studies, as well as some of the trials carried out in the context of the COVID-19 pandemic [14,15] showed that the higher reactogenicity of RNA vaccines respect to what observed for influenza vaccines developed using conventional standard technologies might be an issue for the global acceptance of an RNA-based prophylactic vaccine. In the review by Scorza and Pardi, the authors summarize the current state of the art in terms of preclinical and clinical studies, as well as the major roadblocks that need to be overcome for implementation [16]. In this context, they describe the strategies exploited to increase the half-life, stability, cellular uptake and translatability of both self-amplifying and non-replicating mRNA. A distinctive strength of this class of vaccines is their rapid scalable egg-independent production. Of particular interest is the analysis presented in this review benchmarking the strengths and limitations of this class of vaccines in terms of the World Health Organization guidelines for the preferred product characteristics of next generation influenza vaccines [17].

The availability of innovative technologies will certainly positively affect the development of next generation influenza vaccines. However, influenza vaccines based on both traditional and emerging technologies face the major constraint represented by the immune history of the individual vaccinees. The review article from Lewnard and Cobey analyze the specific constraints and provide examples on how the dynamics of immune memory can affect vaccine effectiveness [18]. It is critical to understand the underlying mechanisms driving these processes, which go far beyond the potential impact of the original antigenic sin during immune imprinting. This will allow dissecting differential patterns of vaccine effectiveness in vaccinees depending on immune imprinting and memory. Immediately after birth, human beings lose their naivety, and previous infection and vaccination events condition responsiveness, for good or bad, from season to season, according to age and the antigen contact history.

## Outlook:

The rational design of next generation influenza vaccines able to confer protection in all age and vulnerable groups is an ambitious and challenging venture. Different approaches can be exploited to stimulate the production of broadly protective vaccine responses [19]. Among them, the more conserved structures of the virus are used as an antigenic target, such as the stalk region of the hemagglutinin, or the matrix protein 2, which is highly conserved between influenza subtypes. An alternative approach relies on the exploitation of in silico tools to develop consensus sequences for computationally optimized broadly reactive hemagglutinin antigen variants (COBRA), which are able to stimulate broadly reactive antibodies [20]. The use of some of these approaches might require the development of alternative tools to assess vaccine efficacy. Current surrogated correlates of protection, which are accepted for the standard influenza vaccines, may not be relevant for next generation vaccines. In fact, the stimulated clearance mechanism might go far beyond traditional neutralization by blocking the virus binding to the receptor (e.g., the inhibition of viral membrane fusion and maturation, antibody-dependent and -independent cytotoxicity, the inhibition of viral replication or spreading).

Interestingly, the adjuvantation of conventional influenza vaccines has also proven effective, not only for improving responsiveness in certain subpopulation groups [21], but also at broadening responsiveness to other influenza subtypes [22,23]. A critical aspect for the development of a vaccine conferring broad cross-protective immunity against influenza is the determination of vaccine efficacy. The clinical trials required to achieve this goal may prove a roadblock. However, the availability of controlled human infection models for the influenza virus is an effective alternative to address this issue [24]. Whether these approaches can lead to a true universal influenza vaccine or to next generation vaccines with a broader efficacy profile, rendering possible to boost against seasonal influenza every 5–10 years instead of every year, or to be prepared for a broad range of strains with a potential to cause pandemics, remains to be elucidate. However, the crystallization of any of these two scenarios will represent a significant improvement with respect to the current situation.

It is important to highlight that regardless of the fact that upcoming next generation vaccines are formulated with well known standard antigens or innovative components promoting cross-protective immunity, or that they will be based on well established or emerging technology platforms for vaccine formulation or delivery, the key problem in vaccine-mediated immunity against influenza will remain. Namely, key influenza-specific issues of interference due to pre-existent immunity and overall poor responsiveness to vaccines in vulnerable individuals (e.g., the elderly, the very young, those affected by co-morbidities) often result in unpredictable poor responsiveness to vaccination. Therefore, the problem of poor protection against influenza infection post vaccination is, by and large, defined by a knowledge gap in key aspects of human immunology, such as the underlying mechanisms involved in poor individual responsiveness, and how to tailor vaccines according to the needs of subpopulation groups to generate broadly protective and long-lasting immunity.

This current knowledge gap can be addressed by performing comprehensive and standardized profiling at baseline and post immunization of responders and non-responders to influenza vaccination in different age groups, both in healthy individuals as well as in those affected by co-morbidities known to affect responses to vaccination. The in-depth analysis and comparison of the obtained immunological and molecular signatures using artificial intelligence tools will enable the identification of the putative underlying mechanisms. These mechanisms will provide intervention targets to develop more efficient tailored vaccination strategies, as well as potential predictive biomarkers of responsiveness to develop innovative diagnostics for the stratification and follow-up of vaccinees.
